# Haemodynamic effects of phenylephrine commenced prior to induction of anaesthesia in older patients undergoing high-risk vascular surgery

**DOI:** 10.1186/cc13354

**Published:** 2014-03-17

**Authors:** H Araujo, R Campbell, D Green, E Mills

**Affiliations:** 1 King's College London, UK; 2 King's College Hospital NHS Foundation Trust, London, UK; 3 LiDCO Ltd, London, UK

## Introduction

Fall in mean arterial blood pressure (MAP) during anaesthetic induction is common and may result in profound hypotension. Using the LiDCORapid (LiDCO Ltd, UK), we previously demonstrated that the fall in MAP is usually driven by a reduction in stroke volume (SV) that was presumed due to venodilation. Low- dose phenylephrine (PE), a venoconstrictor, commenced immediately prior to induction ameliorated these effects to a significant degree [1]. However, it is important that the pre-induction administration of PE should not cause any marked changes in MAP and CO. We retrospectively studied a group of 40 high-risk patients about to undergo major peripheral vascular surgery where PE was commenced immediately pre-induction. The haemodynamic effects of this dose of PE on MAP, SV and CO were analysed.

## Methods

A radial artery line was inserted prior to induction of anaesthesia, and baseline MAP, CO and SV were obtained using the LiDCORapid. The PE infusion was commenced at a rate of 1 to 2 mg/ hour. Remifentanil was then administered using a target-controlled infusion pump until a 2 ng/ml predicted effect site concentration (Ceff) was reached. Propofol was then administered to induce anaesthesia. Haemodynamic changes from the pre-PE baseline to commencement of propofol were recorded at 5-second intervals. The changes in MAP, SV and CO were assessed as the percent change in values obtained immediately prior to PE and followed for at least 5 minutes until commencement of propofol.

## Results

Patient demographics, mean (range): age 71 (46 to 89) years, 31 male, weight 80 (55 to 115) kg, ASA 3 (2 to 4). The changes in MAP and CO were not clinically or statistically significant. MAP increased by an average of only 2%, range -25 to +12; CO by 0%, range -23 to +16 (*P *= 0.35 and *P *= 0.36 respectively).

## Conclusion

In a previous study [1], PE administered prospectively prior to induction markedly reduced the haemodynamic changes following induction of anaesthesia in high-risk patients using remifentanil and propofol. This effect was achieved without significant changes in MAP and CO in the 5-minute to 10-minute period between commencement of PE and induction of anaesthesia.
